# Psychosocial work environment, job mobility and gender differences in turnover behaviour: a prospective study among the Swedish general population

**DOI:** 10.1186/1471-2458-14-605

**Published:** 2014-06-14

**Authors:** Mia Söderberg, Annika Härenstam, Annika Rosengren, Linus Schiöler, Anna-Carin Olin, Lauren Lissner, Margda Waern, Kjell Torén

**Affiliations:** 1 Department of Occupational and Environmental Medicine, Institution of Medicine, Sahlgrenska Academy, University of Gothenburg, Box 414, 405 30, Gothenburg, Sweden; 2 Department of Sociology and Work Science, University of Gothenburg, Box 100, 405 30, Gothenburg, Sweden; 3 Institute of Medicine, Sahlgrenska Academy, University of Gothenburg, Gothenburg, SU/Östra, 416 85, Sweden; 4 Department of Public Health and Community Medicine, Sahlgrenska Academy, University of Gothenburg, Box 414, 405 30, Gothenburg, Sweden; 5 Section of Psychiatry and Neurochemistry, Institution of Neuroscience and Physiology, Sahlgrenska Academy, University of Gothenburg, Blå Stråket 15, 41345, Gothenburg, Sweden

**Keywords:** Job demand-control, Effort-reward imbalance, Job mobility

## Abstract

**Background:**

Throughout the literature, substantial evidence supports associations between poor psychosocial work characteristics and a variety of ill-health outcomes. Yet, few reports strategies workers carry out to improve detrimental work conditions and consequently their health, such as changing jobs. The aim of this study was to examine if adverse psychosocial work exposure, as measured with the job demand-control and effort-reward imbalance models, could predict job mobility over a 5 years observation period.

**Method:**

Participants were working men and women (n = 940; 54.3% women), aged 24–60 years from the population of Gothenburg and surrounding metropolitan area. Job demand-control and effort-reward variables were compared with independent t-tests and chi2-test in persons with and without job mobility. Multivariate logistic regression was used to analyse whether psychosocial factors could predict job mobility. All regression analyses were stratified by gender.

**Results:**

Exposure to a combination of high demands-low control or high imbalance between effort and reward was related to increased odds of changing jobs (OR 1.63; CI 1.03-2.59 and OR 1.46; CI 1.13-1.89 respectively). When analysing men and women separately, men had a higher OR of changing jobs when exposed to either high demands-low control (OR 2.72; CI 1.24-5.98) or high effort-reward imbalance (OR 1.74; CI 1.11-2.72) compared to reference values. The only significant associations for women was slightly decreased odds for turnover in high reward jobs (OR 0.96; CI 0.92-0.99).

**Conclusions:**

The results indicate that workers will seek to improve poor work environment by changing jobs. There were notable gender differences, where men tended to engage in job mobility when exposed to adverse psychosocial factors, while women did not. The lack of measures for mechanisms driving job mobility was a limitation of this study, thus preventing conclusions regarding psychosocial factors as the primary source for job mobility.

## Background

Modern work implies exposure to different quality of work environment. According to Statistics Sweden [1] about 66% of the Swedish working population perceive their work situation as stressful and 42.5% that work was too psychologically demanding. Substantial evidence supports associations between impairing psychosocial work characteristics and a variety of ill-health outcomes; such as cardiovascular and ischemic heart disease [2-4], mental health disorders [5-8] and musculoskeletal problems [9,10]. Despite these illustrated relationships few studies examine strategies workers carry out to improve poor psychosocial job conditions and consequently their health, such as job mobility.

In the context of evaluating psychosocial work environment, there are two predominant models; the job demand-control structure (JDC) [11,12] and the effort-reward imbalance model (ERI) [13]. Job demand captures psychological work load and job control to what extent the individual can influence content and volume of work tasks. Commonly these two variables are dichotomized into high/low and combined according to Karasek [11], high strain (*high demand-low control*), active (*high demand-high control*), passive (*low demand-low control*) and low-strain (*low demand-high control*). As for the ERI model, effort is similar to job demand and measures work intensity, while reward evaluates perceived esteem from colleagues and management, adequate salary and job security. Both high strain and reciprocity between effort spent and reward received has frequently been linked to poor physical and psychological health [2-10].

Studies examining relationships between mentioned models and job mobility are sparse, predominantly performed among health care workers and lack gender stratified analyses. Further, many studies evaluate turn-over *intention*, rather than actual job mobility. Although both job turnover and the intention to leave an employment are steps in the job mobility process, they differ qualitatively. The intention to leave is the preceding attitude towards possible turnover, whereas job mobility is the executed behaviour. The temporal aspect of the study design is also important since cross-sectional studies can only evaluate an intention, while longitudinal studies can capture the followed through behaviour.

Some few longitudinal studies have found that high strain [14] and low job control [15] among blue-collar workers predicted executed job mobility and that nurses experiencing high ERI reported intention to leave their employment at a 1-year follow-up [16]. Yet another paper, examining JDC or ERI as predictors for intention to leave the nursing profession, displayed association for ERI as a predictor for turnover intention, but not for JDC [17]. A handful of cross-sectional studies in samples consisting of health care workers have illustrated that both high strain [18,19] and high ERI [20] could be linked to the intention to leave current organization.

Additionally, some paper have highlighted health consequences from being “locked-in” at work, meaning a combination of poor work environment and reduced possibilities for job mobility. One report showed that the combination of high ERI exposure and being locked-in at work is related to long-term sick leave [21]. Another study [22] illustrated that workers experiencing locked-in positions had delayed return to work and more often reported mental ill-health, than subjects who could change jobs. It has also been shown that similar psychosocial factors could predicted both job mobility and prolonged sick leave among nurses [23], thus emphasising job mobility as an important factor in the context of health beneficial strategies among workers.

The aim of this study was to examine if psychosocial work factors, measured with job demand-control and effort-reward imbalance could predict job mobility. Our study has a longitudinal design with participants drawn from the general population and thus can evaluate actual job mobility among the general workforce. None of the previous papers found analysed men and women separately; therefore our analyses were stratified by gender to detect possible dissimilarities.

## Method

### Sample

**Figure 1:**
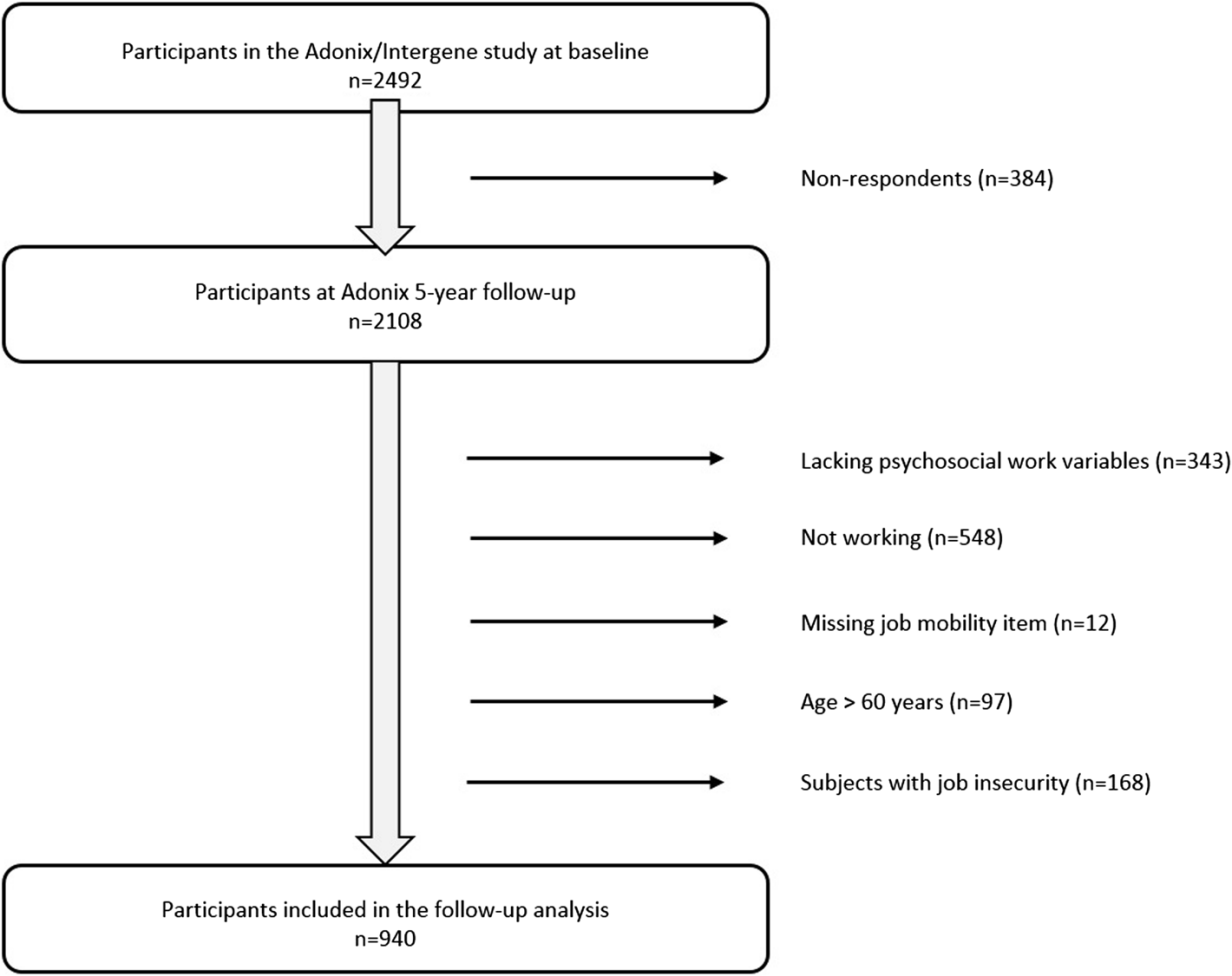
Flowchart illustrating exclusion of participants from analysed sample.

Baseline data collection was part of the INTERGENE and ADONIX projects. In brief, participants aged 24–74 years were randomly selected from the source population of Gothenburg and surrounding metropolitan area. At baseline all subjects were mailed participant information, two questionnaires and a clinical examination invitation. A supplementary questionnaire was administrated during the examination. Detailed study methodology has been published elsewhere [24,25]. A total of 2492 subjects accepted participation at baseline. Five years later all baseline participants were sent the ADONIX follow-up questionnaire. Out of those, 2108 individuals replied (54.4% women).Since analyses were based on work variables, all subjects who had not completed the psychosocial questionnaire (n = 343), were not working (n = 548) or had not filled-in job mobility items in the follow-up questionnaire (n = 12) were excluded. Additionally, subjects aged over 60 (n = 97) were not included, because reported job change in that age group predominantly referred to retirement. Further, persons with “yes” responses to the Effort -Reward Imbalance at Work Questionnaire item “Are you at risk of losing your job?” (n = 168) were excluded as involuntary job mobility might deflate associations between psychosocial exposure and job mobility. The final sample analysed for job mobility thus consisted of 940 subjects (54.3% women). A flowchart illustrating exclusion of participants is found in Figure 1. Both studies have been performed in compliance with the Helsinki Declaration and were approved by the Regional Ethical Review board of Gothenburg.

### Questionnaires

Baseline data was collected with three questionnaires. Two of those questionnaires were sent by mail with the study invitation, one focused on diet and lifestyle and the other was concentrated towards asthma and allergy symptoms. Used data from these two surveys was limited to basic demographics; age, gender, education, civil status and occupation. The third questionnaire, administrated during the baseline clinical examination, contained this study’s main variables; psychosocial work measures. The ADONIX 5-year follow-up questionnaire focused on asthma and allergy disease, but also contained the job mobility item.

#### ***Job demand control***

The job demand-control items were based on a remodelled version [26] of Karasek & Theorell’s Job demand-control structure. Demand and control were explored with three items each, with a scale (1–5) ranging from “Never” to “Almost all of the time”. Sample item for job demands was; “How often during the last year has there been an increased amount of work?” and for job control; “Do you have the possibility to decide your work tasks”. Internal consistency measured by Cronbach alpha was acceptable for both job demand and control (0.7 and 0.8 respectively). The demand and control measures were tallied separately. Demand and control sums each ranged between 3 and 15 and the median for both variables were 11. Each variable was then dichotomized into high and low, using the median of the distribution as cut-off. The dichotomized variables were then combined into categories: *high strain* (high demand-low control), *active* (high demand-high control), *passive* (low demand-low control) and *low strain* (low demand-high control)*.*

#### ***Effort-reward imbalance***

Effort-Reward Imbalance was measured with a standard instrument, the Effort-Reward Imbalance at Work Questionnaire [13]. When using this instrument the item “My work is physically demanding” is usually excluded if the sample consists of a majority of white-collar workers. Participant’s occupation was captured with one item; “What is your occupation?” and classified according to the International Classification of Occupations (ISCO-88) [27]. The classification clearly illustrated that the sample constituted of a majority of white-collar workers. Consequently, the item measuring physical efforts was excluded from the analysis.

The tallied effort scores ranged from 5–25 (mean = 12.3) and sum reward scores ranged between 11 and 55 (mean = 47.9). Cronbach alpha for both effort and reward were 0.7, hence internal consistency was satisfactory. According to praxis, a ratio was created (∑_effort_/((∑_reward × 0.4545)_). The correction factor (0.4545) is used to compensate for the larger number of reward items. The ERI-ratio ranged 0.2-2.0, with mean value of 0.6 (SD 0.3).

In order to better interpret results from the multiple logistic regression we decided to specify levels for the ERI-ratio. Sometimes the ERI-ratio distribution is categorized by the quartiles; however, in our sample the distribution was highly skewed towards lower scores. Additionally, ratio scores above 1.0 is a standard cut-off to indicate a high ERI and since the upper quartile cut-point in our sample was 0.7 a quartile division would be misleading. Instead we decided to specify levels for the ERI-ratio per 0.5 of the distribution.

#### ***Job mobility***

Job mobility was measured with a single item, “Have you changed jobs in the last 5 years?” with a dichotomous response option (yes/no).

### Statistical analysis

Statistical calculations were carried out with SAS version 9.2 for Windows (SAS Institute; Cary; NC). Since the job demand, job control, effort and reward variables were analysed using tallied scores, all missing items were imputed. Imputed values were based on each participant’s mean scores of the remaining items in each variable.

To determine which confounders to include in our model, we utilized purposeful selection of variables for logistic regression as proposed by Hosmer and Lemeshow [28]. Cut-off for variable inclusion throughout the selection procedure was Wald p-value <0.25, with separate calculations for JDC and ERI as main independent variables and with job mobility as outcome. For our multiple logistic regression analysis we considered the following confounders; age, occupational status, civil status, locus of control and depressive symptoms. The purposeful selection process began with univariate analyses of each variable. Any variable meeting the inclusion cut-off was selected as a candidate for multivariate analysis. These variables together with the psychosocial measure constituted the full model. Chosen variables were then entered together in a logistic regression where all variables not meeting p-value <0.25 were excluded. After concluding this step, any variable not selected were added back one at a time and reinserted into the model if meeting inclusion criteria. The remaining variables constituted the reduced model. The main effects between the reduced and full models were then compared. Since changes in main effects for models including either JDC or ERI were both less than 15%, the reduced model was kept.

Our final models consisted of main psychosocial variables with age and occupational status as confounders. Age was entered as a continuous variable. Occupational status was measured with the subject’s occupation at baseline and then classified according to ISCO-88 [27] on a four digit level. Three main occupational categories were then created; *high skilled white-collar* (e.g. executives, managers, professionals), *low skilled white-collar* (sometimes referred as pink-collar work due to constituting of female dominated occupations e.g. office workers, service workers, nurses) and *blue-collar workers* (e.g. plant and machine operators, and jobs without formal training).

All independent variables were then examined for co-linearity with Spearman correlations analyses, due to the categorical properties for some of the variables. None of the paired variables entered displayed a correlation coefficient >0.4 hence they all remained in our analyses. Differences in psychosocial variables between those with and without job mobility were examined with independent t-test for continuous variables and chi2-tests for categorical measures.

Multivariate logistic regression models were then engaged to investigate JDC or ERI variables as predictors for job mobility. Each single variable, job demands, job control, effort and reward, were analysed in separate models. When calculating JDC groups, low strain was used as reference value. In the regression model analysing ERI-ratio, specified levels per 0.5 of the ratio distribution was set. To illustrate possible impact of confounders, both unadjusted models and models controlled for age and occupational status as confounder were calculated. Significance level was p-value 0.05.

## Results

Basic demographics of the sample; age, civil status, level of education and occupational status are presented in Table 1. Descriptives of psychosocial variables (Table 2) illustrated that the majority of participants worked in passive or low strain jobs. The largest proportion of men experienced low strain environment and women most frequently worked in passive jobs. There was also a larger share of women reporting high ERI work environments than men.

**Table 1:** Basic demographics at baseline

	**Men**	**Women**	**Men and women**
**Number of participants, N (%)**	429 (45.6)	511 (54.4)	940 (100)
**Age, Mean (SD)**	45.4 (10.0)	44.4 (9.9)	44.9 (9.9)
**Civil status, N (%)**			
Single	54 (12.7)	64 (12.6)	118 (12.7)
Married/cohabitant	347 (81.7)	375 (73.8)	722 (77.4)
Separated/widow/widower	24 (7.8)	69 (12.2)	93 (10.0)
**Education (highest attended), N (%)**			
Primary school	40 (9.4)	33 (6.5)	73 (7.8)
Lower Secondary	95 (22.3)	112 (22.0)	207 (22.1)
Upper secondary	126 (29.6)	123 (24.1)	249 (26.6)
University/College	165 (38.7)	242 (47.5)	407 (43.5)
**Occupational Status, N (%)**			
*High skilled white-collar work*			
1. Executive work	51 (12.1)	30 (6.0)	81 (8.8)
2. High skilled academic	106 (25.1)	139 (27.6)	245 (26.5)
3. Low skilled academic	85 (20.1)	110 (21.9)	195 (21.1)
*Low skilled white-collar*			
4. Client/office service work	35 (8.3)	96 (19.1)	131 (14.2)
5. Care/retail service work	19 (4.5)	97 (19.3)	116 (12.5)
*Blue-collar work*			
6. Farming, gardening, foresting	4 (1.0)	2 (0.4)	6 (0.7)
7. Construction/installation work	67 (15.8)	2 (0.4)	69 (7.5)
8. Machine op/transport	39 (9.2)	9 (1.8)	48 (5.2)
9. Job w/o formal training	16 (3.8)	17 (3.4)	33 (3.6)

**Table 2:** Descriptives of psychosocial work environment variables at baseline

	**Men**	**Women**	**Men and Women**
Job demand-control, mean (SD)			
Demand	10.5 (2.3)	10.7 (2.2)	10.6 (2.2)
Control	11.5 (3.0)	10.4 (3.0)	10.9 (3.0)
Job demand-control groups, N (%)			
High strain	57 (14.4)	119 (25.1)	176 (20.0)
Active	83 (20.4)	59 (12.4)	142 (16.1)
Passive	116 (28.6)	173 (36.4)	289 (32.8)
Low strain	150 (37.0)	124 (26.1)	274 (31.1)
Effort-reward imbalance, mean (SD)			
Effort	12.4 (4.4)	12.2 (4.7)	12.5 (4.6)
Reward	50.0 (5.6)	48.1 (5.9)	48.9 (5.8)
Effort-reward imbalance, N (%)			
Low ERI (ratio scores <1.0)	388 (95.6)	438 (91.4)	826 (93.3)
High ERI (ratio scores >1.0)	18 (4.4)	41 (8.6)	59 (6.7)

Differences in psychosocial variables at baseline between those who had changed jobs and those who did not at the 5-year follow-up can be found in Table 3. The majority of the participants remained at the same job (74.5%). Subjects with job mobility had significant higher job demands, lower control, lower rewards and higher ERI-ratio than those who remained. When examining men and women separately, a slightly larger percentage of women changed jobs (26.8%) than men (24.0%). Further, men with job turnover displayed higher demands and lower control than other male subjects. For women, reward at baseline was lower among those who had changed jobs.

**Table 3:** Differences between subjects with and without job mobility

	**Job mobility**	**Remained at same job**	
**Number of participants, N (%)**			
All	240 (25.5)	700 (74.5)	
Men	103 (24.0)	326 (76.0)	
Women	137 (26.8)	374 (73.2)	
	**Job mobility**	**Remained at same job**	**p-value**
**Job demand, mean (SD)**			
All	10.9 (2.2)	10.5 (2.3)	0.02
Men	11.0 (2.3)	10.4 (2.2)	0.03
Women	10.9 (2.1)	10.6 (2.3)	0.27
**Job control, mean (SD)**			
All	10.5 (3.2)	11.1 (3.0)	0.01
Men	10.8 (3.2)	11.7 (2.9)	0.009
Women	10.2 (3.2)	10.5 (3.0)	0.32
**Job demand-control exposure, N (%)**			
High strain			
All	49 (22.9)	127 (19.0)	0.44
Men	19 (20.7)	124 (39.5)	0.08
Women	30 (24.6)	89 (25.1)	0.65
Active			
All	34 (15.9)	108 (16.2)	0.64
Men	18 (19.6)	65 (20.7)	0.58
Women	16 (13.1)	43 (12.2)	0.95
Passive			
All	73 (34.1)	216 (32.4)	0.90
Men	29 (31.5)	87 (27.7)	0.48
Women	44 (36.1)	129 (36.5)	0.62
Low strain			
All	58 (27.1)	216 (32.4)	0.04
Men	26 (28.3)	124 (39.5)	0.02
Women	32 (26.2)	92 (26.1)	0.77
**Effort, mean (SD)**			
All	13.0 (4.7)	12.3 (4.5)	0.06
Men	13.2 (4.6)	12.4 (4.4)	0.11
Women	12.8 (4.8)	12.2 (4.7)	0.27
**Reward, mean (SD)**			
All	47.9 (6.2)	49.3 (5.7)	0.003
Men	49.3 (6.0)	50.1 (5.5)	0.22
Women	46.9 (6.2)	48.5 (5.7)	0.01
**ERI-ratio, mean (SD)**			
All	0.62 (0.27)	0.57 (0.25)	0.02
Men	0.61 (0.24)	0.56 (0.23)	0.08
Women	0.62 (0.28)	0.58 (0.27)	0.12

Logistic regression analyses examining single psychosocial variables as predictors for job mobility displayed in Table 4. There were only minor increased odds ratio for changing jobs in the unadjusted models if exposed to high job demands (OR 1.09; CI 1.01-1.17) and high effort (OR 1.04; CI 1.00-1.07), and slightly lowered odds for job turnover for high job control (OR 0.93; CI 0.88-0.98) and rewards (OR 0.96; CI 0.94-0.99). The results for the adjusted model displayed similar results, with the exception for effort as job mobility predictor which became non-significant. When stratifying by gender, unadjusted analysis among men illustrated somewhat elevated odds ratio for job mobility in high demand work and lowered odds for high control. Results among female illustrated that high rewards was related to decreased odds for job mobility.

**Table 4:** Multiple logistic regression analysis between single psychosocial variables and job mobility (OR = Odds ratio, CI 95% = 95% confidence interval)

	**Men**	**Women**	**Men and women**
	**OR (CI 95%)**	**OR (CI 95%)**	**OR (CI 95%)**
	**p-value**	**p-value**	**p-value**
MODEL 1*			
Job demands	1.13 (1.01-1.25)	1.06 (0.96-1.16)	1.09 (1.01-1.17)
	0.03	0.26	0.02
Job control	0.89 (0.83-0.96)	0.97 (0.90-1.03)	0.93 (0.88-0.98)
	0.004	0.30	0.005
Effort	1.05 (0.99-1.11)	1.03 (0.98-1.07)	1.04 (1.00-1.07)
	0.09	0.24	0.05
Reward	0.97 (0.93-1.01)	0.96 (0.92-0.99)	0.96 (0.94-0.99)
	0.17	0.01	0.003
MODEL 2**			
Job demands	1.12 (0.99-1.26)	1.08 (0.98-1.19)	1.10 (1.02-1.18)
	0.05	0.27	0.01
Job control	0.87 (0.80-0.96)	0.95 (0.89-1.03)	0.92 (0.87-0.97)
	0.004	0.19	0.002
Effort	1.03 (0.97-1.09)	1.03 (0.99-1.08)	1.03 (0.99-1.07)
	0.37	0.18	0.10
Reward	0.97 (0.93-1.02)	0.95 (0.92-0.99)	0.96 (0.93-0.99)
	0.24	0.007	0.003

Each variable job demand, job control, effort and reward were analysed in separate models.

*Unadjusted.

**Adjusted for age and occupational status.

The logistic regression analyses for the combined job demand-control variables as job mobility predictors (Table 5) did not result in any significant associations in the adjusted model, except when stratifying by gender. These analyses showed that among men, odds ratio for job mobility more than doubled when exposed to high strain, compared to males in low strain jobs (OR 2.52; CI 1.25-5.01). Calculations adjusted for age and occupational status resulted in increased odds ratio for changing jobs when exposed to high strain, both when analysing all sample subjects (OR 1.3; CI 1.03-2.59) and for male participants (OR 2.52; CI 1.24-5.98). All relationships between combined job demand-control variables and job turnover among women tested null, regardless if the regression model was adjusted or not.

**Table 5:** Multiple logistic regression analysis between job demand-control and job mobility (OR = Odds ratio, CI 95% = 95% confidence interval)

	**Men**	**Women**	**Men and women**
	**OR (CI 95%)**	**OR (CI 95%)**	**OR (CI 95%)**
	**p-value**	**p-value**	**p-value**
MODEL 1*			
Low strain (ref)	1.00	1.00	1.00
Passive	1.65 (0.91-3.00)	0.99 (0.58-1.68)	1.29 (0.87-1.91)
	0.10	0.84	0.71
Active	1.32 (0.68-2.59)	1.12 (0.55-2.27)	1.20 (0.74-1.95)
	0.42	0.69	0.88
High strain	2.52 (1.25-5.01)	0.98 (0.55-1.75)	1.48 (0.95-2.30)
	0.01	0.83	0.20
MODEL 2**			
Low strain (ref)	1.00	1.00	1.00
Passive	1.80 (0.93-3.46)	1.01 (0.58-1.76)	1.23 (088–2.01)
	0.08	0.96	0.18
Active	1.27 (0.62-2.59)	1.18 (0.57-2.42)	1.22 (0.74-2.01)
	0.52	0.66	0.45
High strain	2.72 (1.24-5.98)	1.14 (0.63-2.07)	1.63 (1.03-2.59)
	0.01	0.67	0.04

*Unadjusted.

**Adjusted for age and occupational status.

High ERI was related to increased odds ratio for changing jobs (Table 6) both in the unadjusted and adjusted analyses (OR 1.42; CI 1.11-1.81 and OR 1.46; CI 1.13-1.89 respectively). The analyses examining men and women separately showed that men displayed elevated odds for job turnover in both models. Similar to calculations with JDC, there were no associations between ERI and job mobility for women.

**Table 6:** Multiple logistic regression analysis between effort-reward imbalance and job mobility (OR = Odds ratio, CI 95% = 95% confidence interval)

	**Men**	**Women**	**Men and women**
	**OR (CI 95%)**	**OR (CI 95%)**	**OR (CI 95%)**
	**p-value**	**p-value**	**p-value**
MODEL 1*			
Effort-reward ratio	1.86 (1.24-2.80)	1.20 (0.88-1.64)	1.42 (1.11-1.81)
	0.003	0.24	0.005
MODEL 2**			
Effort-reward ratio	1.74 (1.11-2.72)	1.31 (0.95-1.81)	1.46 (1.13-1.89)
	0.02	0.10	0.004

*Unadjusted.

**Adjusted for age and occupational status.

## Discussion

Both high demands-low control and high effort-reward imbalance predicted job mobility in our sample drawn from the general population. Considering the substantial evidence for relationships between psychosocial work characteristics and both physical and mental ill-health outcomes [2-10], it is a positive finding that workers might engage in active strategies to improve their work environment and thereby their health. Notable is that when stratifying by gender all relationships between psychosocial variables and job mobility, except reward, tested null among women. In comparison, men in high strain jobs displayed more than doubled odds for job turnover compared to male subjects in low strain work environment. High ERI was also a predictor for job mobility among men.

Our findings parallel those of earlier studies using intention to leave current job [16,18-20] and executed job mobility [14] as outcome. Low job control as a predictor for turnover [15] was also partly confirmed in this study as results illustrated minor, but significant associations. Our results deviate from one previous study which illustrated that JDC was not related to intention to leave among nurses [17]. It is plausible that the dissimilarities in results stem from differences in job mobility behaviour between this specific occupational category and tendencies in a general population.

Our study did also illustrate noteworthy differences between men and women in job mobility behaviour. Siegrist, the creator of the ERI model, argues that when exposed to poor job conditions workers will usually try to improve negative content before changing jobs [6]. It is possible that women were more proactive and improved their job conditions, thus reducing the need for job change. Unfortunately our study lacked data on job conditions at follow-up; hence this option could not be evaluated.

Another cause for not changing jobs is that negative psychosocial exposures can be endured for future gains [29]. The process is unhealthy, and will be of particular harm if the investment does not pay off. This could mean that women, who in this sample displayed a high percentage of individuals with university education, might have a strong career focus and thus voluntarily remain in adverse job situations. Statistics Sweden [30] does however, report gender disparities in the labour market such as unequal salary and male dominance in high status positions. Thus our study might imply that women more frequently expose themselves to detrimental work conditions that do not pay off, and as a consequent are at greater occupational health risks.

Lack of job mobility can also be caused by a saturated job market. Studies show that the combination of poor psychosocial work environment and limited work options increases mental ill-health [6,23,31], days on sick leave [21,23] and delays return to work [22]. The quality of this process is likely to be different than that which is self-inflicted, since it is involuntary and unlikely lead to future profits. Workers can also be “locked in occupation” [21]. This concept refers to inability to find employment outside current occupation, but also that job mobility within the occupation is unlikely to result in improved work environment. A large proportion of the women in our sample worked in health care/retail service work. Health care occupations are among the most stressful in Sweden today [1]. It is possible that females in this occupational group are locked in their profession, which could further explain gender dissimilarities in turnover behaviour.

### Limitations

One major limitation in our study was that we could not elucidate motives for job mobility. If job changes are due to career opportunities or redundancies, psychosocial stressors have less importance when deciding to change jobs. This shortage may therefore have blurred to what extent psychosocial poor work exposure was related to job mobility. This might be particularly the case for women, who had a slightly higher percentage of job mobility than men, yet their turnover were not related to psychosocial factors. Hence, we cannot elaborate further is women’s job mobility is more related to issues such as career strategies or short-term employments, than psychosocial variables. It would also have been beneficial to investigate intention to leave at baseline in order to investigate whether this variable predicted actual job mobility.

A further limitation was the constitution of the studied sample. A previous selection bias study [32] illustrated that individuals who declined participation in the Intergene and Adonix studies tended to be men, younger, have lower education and to have been born outside Scandinavia. Drop-out of male subjects with lower education might have skewed the male proportion of sample towards individuals with higher occupational status and therefore better labour market opportunities. This could possibly also have inflated the odds ratio for the association between poor psychosocial work environment and job mobility among men. The overall drop out of both younger subjects and individuals with lower education could also have enhanced odds ratio for job mobility when exposed to poor psychosocial job environment, since older established individuals and those with higher education generally have better labour market opportunities.

## Conclusions

This study provides additional evidence for poor psychosocial work environment as one possible predictor for job mobility. Results also showed differences in turnover behaviour between men and women. Men tended to change jobs when exposed to adverse job conditions, but not women. Plausible explanations are gender disparities in the labour market and locked-in job situations. The results highlight the importance of job mobility as a health beneficial strategy, but also that women might have more limited labour market options and consequently are more exposed to occupational health related risks. The lack of measures for mechanisms driving job mobility was a limitation of this study, thus preventing conclusions regarding psychosocial factors as the primary source for job mobility. Further studies would benefit from including motivational variables and investigate gender differences.

## Competing interests

The authors declare that they have no competing interests.

## Authors’ contributions

MS is the first author and have drafted and designed the manuscript, carried out the statistical analysis and interpretation of data. AH has made substantial contributions to interpretation of data and reviewing the manuscript, especially regarding gender aspects and health consequences from psychosocial work exposure. AR has made substantial contributions to collection of data, study design, interpretation of data and critically reviewing the manuscript. LS has contributed to study design, made substantial contribution to the statistical analysis, interpretation of data and reviewing the manuscript. AO is the project leader for collection of Adonix follow-up data, has contributed with study design, interpretation of data and reviewing the manuscript. LL is the project leader for the Intergene project and collection of project data, has contributed with study design, interpretation of data and reviewing the manuscript. MW has made substantial contributions to drafting the manuscript, interpretation of data and critically reviewing the manuscript. KT is the project leader for the Adonix project. KT have made substantial contributions to collection of data, study design, interpretation of data, drafting of the manuscript and interpretation of data. All authors agree to be accountable for all aspects of the work in ensuring that questions related to the accuracy or integrity of any part of the work are appropriately investigated and resolved. Additionally, all authors have given final approval of the version to be published and agree to be accountable for all aspects of the work in ensuring that questions related to the accuracy or integrity of any part of the work are appropriately investigated and resolved.

## Pre-publication history

The pre-publication history for this paper can be accessed here:

http://www.biomedcentral.com/1471-2458/14/605/prepub
